# Rapid Method of Inserting and Finishing Contour Fillings without the Aid of a Matrix

**Published:** 1890-03

**Authors:** J. E. Waitt


### RAPID METHOD OF INSERTING AND FINISHING CONTOUR FILLINGS WITHOUT THE AID OF A MATRIX.[1]

BY J. E. WAITT, D.M.D.

For years, science and skill have been coming to that place where each and every department of life is being forced to that point where its work may be accomplished with the greatest ease and in the shortest time. The department which we represent has been among the foremost in devising ways and means for the accomplishment of our daily work with the least inconvenience both to ourselves and our patients. It shall be the aim of this paper to present you, in a few words, a few (possibly new) ideas, and thereby assist you in the labors of the day, and the rapid and satisfactory completion of those large or difficult contour fillings with which we have to deal.

By the use of Dr. Jack's and other matrices very many of the difficult operations in contour filling have become, under the skilful hand, very simple operations; and operations once requiring hours for their completion are now accomplished in minutes.

The impossibility of having the walls of a cavity in full view, and the inconvenience of easily adjusting a matrix, led me to discard all forms of matrices, and look about for a method of quickly reaching the same or a better result. After some years of experimenting with methods, foils, and preparation of cavities, I am pleased to present you a method which you will find to answer the requirements of the case, thereby making operations, formerly long and difficult, so short that they become a pleasure both to the patient and the operator.

First, space by a separator or tape, to the width of a No. 3 or 4 separating file. Apply the dam in the usual or the following manner: In the case of a right superior second bicuspid, lay the dam over the face, and apply it to the mouth in the position it is to occupy, then with a pencil mark the centre of each tooth from the right superior lateral to and over the first molar. Punch out a hole for each tooth, and then apply the dam, beginning at the

---

[1] Read before the American Academy of Dental Science, December 4, 1889.

lateral and working backwards. Over the molar slip a clamp, or, if close to the second molar, pass a ligature up between, and leave it. Then, with a thin and flat burnisher, turn the dam up on itself around each tooth, and dry with cotton.

The dam applied in this manner obviates the tying of a ligature about each tooth, which is a nerve-trying experience. This method allows greater freedom for working, because the dam is out of the way, and also affords better light. It will be found to answer the purpose for large cervical cavities when properly applied.

Next, prepare the cavity as usual, except do not cut any retaining points, but in their place cut a fine groove across the cervical wall and down the buccal and palatal or lingual walls with a fine burr, and make the opening on the crown surface when possible, slightly dovetailing towards the body of the tooth; then, with a sand-paper disk, smooth the mesial or distal surface, as the case may be.

The gold to be used becomes the next feature, and must be a soft gold, easily moulded under a burnisher. I use the new gold of the Boston Dental Manufacturing Company, ropes No. ½. These may be flattened and made into cylinders with great convenience.

In using soft foil, take No. 4 soft, cut in one-half sheets, and roll to about the size of three-sixteenths of an inch diameter, and flatten. Each cylinder to be of a length slightly exceeding the depth of the cavity from front to back, and its size in the square to depend upon the width of the cavity and the method of condensation.

I prefer the No. ½ ropes to any other, for the ease with which cylinders may be made from it. For the intermediate layers, of which I will explain later, the Boston Dental Manufacturing Company's No. 4 foil is the form used.

In preparing the cylinders, take a strip full length of soft gold, grasp it with a pair of pliers, and fold over and over until the size wanted is rolled; then condense easily endways, then sideways, and repeat until it is of the desired square.

Never cut a strip in two lengthways to get a short cylinder, but rather fold it upon itself lengthwise, and proceed to roll as in the usual manner. With many of these cylinders of various sizes rolled during leisure-hours, or by an assistant, considerable time may be saved at the moment of greatest strain to yourself and patient.

Now place a floor of these squared cylinders across the bottom of the cavity, allowing the ends to extend slightly over the edge, and with a large round or foot-shaped plugger partially condense this layer; then, with a couple of pieces of No. 4 foil made cohesive and *single thickness*, placed over the partially condensed surface, the whole is thoroughly condensed with a smaller, round plugger, care being taken not to allow the plugger to enter the gold extending over the edge.

When thoroughly condensed, pass a thin, flat burnisher up by the ends, and, with a slight pressure, force them over the edges of the cavity in each direction, making the burnishing carry the gold over the edges, thereby locking it and preventing tearing it out. Increase the pressure upon the burnisher until the ends are well condensed, and shaped to the desired contour.

When this point is reached, place a couple of thin layers of cohesive gold over the whole, and condense well. This is done for the purpose of locking each layer and giving edge-strength to the contour. Burnish this edge well, and then proceed with your second layer of soft cylinders, as you have done with your first, with the exception that the middle cylinders are to be slightly longer than the side ones, to allow for the swell of the contour. Burnish, and proceed as before.

The crown surface can be finished by building up to the desired shape with cohesive gold, which can always be made to unite with the soft gold, if the first union is made with pieces of a single thickness, well united by mechanical force, and condensed with the soft gold.

Contour fillings, built in this manner, do not need any finishing with a file or sand-paper, as the whole surface is left finished at each stage, and any amount of overlapping gold is burnished off, leaving a clean, smooth edge, and highly polished.

By this method the entire wall edge is constantly in view, and one point is not left for another until the former has been completely *filled* and finished, which alone is enough to commend this method to our careful consideration.

